# Quality assessment reporting checklists for microsimulation models: A scoping review protocol

**DOI:** 10.1371/journal.pone.0344385

**Published:** 2026-03-09

**Authors:** Claire de Oliveira

**Affiliations:** 1 Institute for Health Policy, Management and Evaluation, Dalla Lana School of Public Health, University of Toronto, Toronto, Ontario, Canada; 2 Campbell Family Mental Health Research Institute, Centre for Addiction and Mental Health, Toronto, Ontario, Canada; 3 Institute for Mental Health Policy Research, Centre for Addiction and Mental Health, Toronto, Ontario, Canada; 4 ICES, Toronto, Ontario, Canada; 5 Uppsala Health Economics, Department of Public Health and Caring Sciences, Uppsala University, Uppsala, Sweden; University of Porto Faculty of Medicine: Universidade do Porto Faculdade de Medicina, PORTUGAL

## Abstract

**Background:**

Microsimulation models are computer-based models that can be used to understand how economic agents behave in different situations. These models are used by governments to help them make decisions. However, it is important these models are well built and produce useful information. Reporting checklists can help guide researchers confirm that all the necessary elements are included in a model. There are currently no formal reporting checklists to evaluate the quality of microsimulation models. This protocol aims to describe a scoping review, which will retrieve and synthesise the literature on any existing quality assessment checklists for microsimulation models and/or any literature that provides best practices, guidelines, and/or recommendations around which elements should be included.

**Methods:**

We will undertake a scoping review followed the PRISMA guidelines for Scoping Reviews. We will search MEDLINE, Embase, EconLit, and Web of Science, with an update closer to the time of manuscript submission. In addition, where relevant, we will undertake Google searches and searches on specific journals (e.g., International Journal of Microsimulation) and websites (e.g., https://www.microsimulation.ac.uk/) to complement the database searches. We will extract relevant data on quality dimensions and use a narrative synthesis to describe the recommendations.

**Discussion:**

There are no formal checklists to assess the quality of microsimulation models. Moreover, no scoping reviews have been undertaken on this topic. This work will synthesise any existing recommendations regarding the development of robust microsimulation models. A validated quality assessment reporting checklist will be the first of its kind and thus, fill an important gap in the literature.

## Introduction

In a time of limited health care resources, it is crucial to make informed decisions around resource allocation; to do so, decision makers require robust information to make sound investments. Microsimulation models are computer-based models that can be used to simulate the behaviour of micro-entities such as individuals in response to interventions and/or health policies [1]. Microsimulation models can also be used to test scenarios, which cannot be tested in the real world, such as trials. Thus, as an ex-ante policy evaluation method, microsimulation models are valuable tools to examine potential behavioural and economic effects of interventions and policies and to guide decision-making [1,2]. However, to encourage the uptake of microsimulation models by policy makers, it is important that these models produce valid and robust outputs of high quality. In other words, it is important that the purpose(s) of the model is (are) well-defined, the data employed are appropriate and representative of the population studied, the model is transparent, uncertainty and model validation are considered, and model outputs are generalizable.

Checklists, commonly used in the health economics field, provide researchers with guidance in the reporting and/or quality assessment of a specific type of analysis. These checklists can help improve reporting and its consistency as well as methodological quality can allow readers to assess all important aspects of the study, while facilitating comparison across studies. Currently, there is no formal quality assessment reporting checklist for microsimulation models. Establishing the quality of microsimulation models is important as it can encourage their use by both academics and policy makers [3], the latter of whom are looking for models that produce robust outputs to guide decision making. There are many potentially relevant dimensions of quality to consider when examining microsimulation models; however, it can be challenging to discuss quality in abstract or general terms [3]. The existing reporting guidelines in the health economics field are mainly focused on economic evaluations. For example, the Consolidated Health Economic Evaluation Reporting Standards (i.e., CHEERS) checklist is typically used to evaluate the reporting standards of economic evaluations [4], some of which may make use of microsimulation techniques; however, issues like study perspective and measurement of effectiveness and costs, which are specific to economic evaluations, may not be relevant when assessing microsimulation models, particularly all-purpose, stand-alone microsimulation models. Other reporting checklists, such as the 2014 International Society for Pharmacoeconomics and Outcomes Research (ISPOR) [5] and the 2016 Assessment of the Validation Status of Health-Economic Decision Models (AdViSHE) checklists [6], were mainly developed to examine the credibility and assess the quality of economic models, respectively, and therefore not all elements of these checklists are relevant to microsimulation models. Finally, there is a reporting checklist for discrete event simulations in health care [7]. However, this checklist is specific to discrete event simulations only and, while relevant, it is not directly applicable to other types of simulation models. Moreover, most of these checklists cover reporting standards; while reporting checklists are useful, it is also important to have checklists with a focus on quality standards.

As part of a larger study (i.e., a scoping review of microsimulation models on mental health), a quality assessment reporting checklist for microsimulation models – the Quality Assessment Reporting for Microsimulation Models (QARMM) checklist – was developed [8]. The six main elements of the QARMM checklist are largely based on work done by Sutherland (2018) [3], which describes the necessary dimensions to account for when considering the quality of microsimulation models, as well as the reporting quality checklist developed for discrete event simulations in health care [7]. The six quality assessment criteria are as follows: 1) purpose of the model, 2) data, 3) transparency, 4) uncertainty, 5) validation, and 6) generalizability, where the first three relate to the model development and structure and last three relate to the validity and scope of the results produced by the model. Each item was determined to be worth one point; half points were given in instances where the criterion was not fully met. However, the QARMM checklist has some limitations. First, it was developed specifically for the purpose of assessing the quality of microsimulation models in a scoping review, but it was not validated. Moreover, there are several aspects of the checklist that could be further improved upon. For example, while many checklists score each item equally (such as the CHEERS checklist [4]), there may be some elements that may be more important than others when developing microsimulation models (e.g., validation) and consequently should be assigned more weight (in other words, different weighting criteria should be applied). However, creating different scores for each item requires additional value judgments and the involvement of experts, which was beyond the main purpose of the aforementioned review.

The objective of this protocol is to describe a scoping review whose goal is to retrieve and synthesise the literature on existing quality assessment checklists for microsimulation models (if/where these exist) and/or any literature that provides best practices, guidelines, and recommendations around which elements should be included/accounted for when building microsimulation models.

## Methods

### Study design

Scoping reviews are ideal for describing the broader literature with regards to a theme, whereas traditional systematic reviews are intended to answer a specific, focused question [9]. Therefore, we propose to undertake a scoping literature review to identify any existing work, which includes quality assessment checklists for microsimulation models and/or recommendations regarding elements that should be included in microsimulation models. We will follow the Joanna Briggs Institute Manual for Evidence synthesis to guide the scoping review methodology [9] and the Preferred Reporting Items for Systematic Reviews and Meta-Analyses extension for scoping reviews (PRISMA-ScR) [10] to guide the reporting. The protocol was registered at Open Science Framework (https://osf.io/wcsq9). The review is planned to start on 1 May 2025.

### Eligibility criteria

We will use two concepts to guide our search: concept 1 – microsimulation and concept 2 – checklist. The search terms for concept 1 will include microsimulation, microsimulations, microsimulator, microsimulated, and microsimulat*. The search terms for concept 2 will include checklist, guide, guideline, guidance, quality appraisal, quality assessment, reporting/publishing template, principle, good/best practice, recommendation, standard, requirement, and instruction, informed by prior, related work [11]. Only studies published in English will be considered as this will be the common language among the reviewers. However, to ensure that a large range of relevant studies are identified, we will not apply any restrictions on publication type, study type, or year limits.

### Search strategy

We will search the following databases since database inception: Ovid MEDLINE, Embase, APA PsycINFO, EconLit, IEEE Xplore, and Scopus, as done elsewhere [11]. We will explore the possibility of searching grey literature as well, if/where possible, by undertaking Google searches (using Google and Google Scholar) and targeted searches on specific journal websites (e.g., International Journal of Microsimulation) and other relevant websites (e.g., https://www.microsimulation.ac.uk/). The lead investigator (CdO) and a research librarian developed a structured search strategy for Ovid MEDLINE reflecting the inclusion and exclusion criteria; this will then be tailored to each database (the full search strategy for Ovid MEDLINE can be found in the appendix). The searches will be re-executed before the final analysis to ensure that the most recently published research is captured in the scoping review. A preliminary search in PubMed produced 399 records; a cursory glance at the titles and abstracts suggests that at least two records may be of potential interest.

### Study selection

Search results will be uploaded onto Covidence, an online systematic review software [12]. Following this, duplicate records will be removed. Once all titles and abstracts are reviewed, all articles deemed relevant will be retrieved for full text review. Titles and abstracts and full text review will be undertaken by two independent reviewers; both reviewers will address disagreements, if/where they arise.

### Data extraction

A data extraction form will be developed and include the following information: author(s), year of publication, article title, journal, method of development of checklist (if/where applicable), proposed quality dimension(s) and/or recommendations that should be considered when developing a microsimulation model, and the validation process of the checklist (if/where relevant). Further elements may be extracted, as needed. The data extraction form will be piloted with two studies by one reviewer before full data extraction and data extraction headings will be revised, if/where required, in discussion with another reviewer. One reviewer will extract and code the data, and a second reviewer will review each entry for accuracy and consistency.

### Data synthesis

The publication selection process will be described using a PRISMA flow diagram [13]. A narrative synthesis will be undertaken to synthesise the existing evidence [14], with particular attention paid to the quality dimensions identified by other researchers as elements that should be included when developing/designing microsimulation models. One reviewer will undertake the data synthesis. Tables summarising details of the extracted data will be developed, where suitable. The evidence will then be used to create a checklist of items that should be followed when developing/designing microsimulation models.

## Discussion

There are likely no existing formal checklists to assess the quality of microsimulation models, beyond the QARMM checklist [8]. Moreover, no scoping reviews have been undertaken on this topic. A validated quality assessment reporting checklist will be the first of its kind. Thus, this work would fill an important gap in the literature and help inform the development of a new quality reporting checklist devoted specifically to microsimulation models. The development and validation of a quality assessment reporting checklist (and, in turn, general guidance on how to develop and design robust microsimulation models) is likely to greatly improve the quality of future microsimulation models. Thus, it is expected that the development and use of this checklist will have an impact on health care policy/decision making.

Findings from the scoping review will be presented at relevant conferences and published in an appropriate peer-reviewed academic journal. In addition, this information will be used to apply for a grant to develop a refined quality assessment reporting checklist for microsimulation models, building off the results obtained from the scoping review. Any relevant changes/amendments to the scoping review, where applicable, will be noted and justified and subsequently recorded in the study protocol.

## Supporting information

S1 File Preferred Reporting Items for Systematic reviews and Meta-Analyses extension for Scoping Reviews (PRISMA-ScR) checklist. (PDF)

S2 File Preferred Reporting Items for Systematic review and Meta-Analysis Protocols (PRISMA-P) 2015 checklist. (DOCX)

S3 File Search strategy. (DOCX)
