# Supplementary material for: Quality assessment reporting checklists for microsimulation models: A scoping review protocol
Source: PLoS One. 2026 Mar 9;21(3):e0344385. doi: 10.1371/journal.pone.0344385 (PMC12970866; doi:10.1371/journal.pone.0344385)
Supplement: S3 File — (DOCX) [file pone.0344385.s003.docx]

**Appendix**

Search strategy for Ovid MEDLINE

| Ovid MEDLINE(R) ALL <1946 to May 22, 2025> | |  |
| --- | --- | --- |
|  |  |  |
| 1 | microsimulat*.ab,hw,kf,ti,kw,id,jw. | 2331 |
| 2 | micro-simulat*.ab,hw,kf,ti,kw,id,jw. | 230 |
| 3 | ("microsim" or "microsims" or "micro-sim" or "micro-sims").ti,ab. | 17 |
| 4 | (("MSM" or "MSMs") and (microsimulat* or micro-simulat*)).ti,ab. | 20 |
| 5 | (microanalytic* adj3 (model* or simulat* or method$1)).ab,hw,kf,ti. | 190 |
| 6 | (micro-analytic* adj3 (model* or simulat* or method$1)).ab,hw,kf,ti. | 33 |
| 7 | (microscopic* adj3 (model* or simulat* or method$1)).ti. | 973 |
| 8 | (microscopic* adj3 (model* or simulat* or method$1)).ab,hw,kf. /freq=2 | 483 |
| 9 | (((individual* adj1 level*) or (micro adj1 level*)) adj3 (model* or simulat* or method$1)).ab,hw,kf,ti. | 1778 |
| 10 | agent* based model*.ab,hw,kf,ti. | 3861 |
| 11 | agent* based simulat*.ab,hw,kf,ti. | 778 |
| 12 | individual level model*.ab,hw,kf,ti. | 138 |
| 13 | individual level simulat*.ab,hw,kf,ti. | 44 |
| 14 | micro level model*.ab,hw,kf,ti. | 12 |
| 15 | micro level simulat*.ab,hw,kf,ti. | 3 |
| 16 | or/1-15 [**Microsimulations] | 10222 |
| 17 | Checklist/ | 9709 |
| 18 | guideline/ | 16416 |
| 19 | checklist*.ab,hw,kf,ti,kw,id. | 70308 |
| 20 | check list*.ab,hw,kf,ti,kw,id. | 3605 |
| 21 | best practice*.ab,hw,kf,ti. | 45546 |
| 22 | good practice*.ab,hw,kf,ti. | 7563 |
| 23 | recommendation*.ab,hw,kf,ti. /freq=2 | 91957 |
| 24 | guideline*.ab,hw,kf,ti. /freq=2 not (screening guideline* or clinical guideline* or practice guideline* or treatment guideline*).ab,hw,kf,ti. | 93690 |
| 25 | ((standard$1 or policy or policies or instructions or method* or requirement$1) adj3 (microsimulat* or micro-simulat* or "microsim" or "microsims")).ab,hw,kf,id,kw. /freq=2 | 8 |
| 26 | (protocol* adj3 (microsimulat* or micro-simulat* or "microsim" or "microsims")).ab,hw,kf,ti. | 1 |
| 27 | (quality adj3 (assess* or evaluat* or apprais*)).ab,hw,kf,ti. | 180508 |
| 28 | (calibrat* adj1 (microsimulat* or micro-simulat* or "microsim" or "microsims")).ab,hw,kf,ti. | 11 |
| 29 | ((develop* or creat* or evaluat*) adj3 (microsimulat* or micro-simulat* or "microsim" or "microsims")).ab,hw,kf,ti. | 396 |
| 30 | evidence based.ab,hw,kf,ti. | 241422 |
| 31 | or/17-30 [**Checklists/guidelines/standards] | 677143 |
| 32 | 16 and 31 [**Microsimulations and checklists] | 698 |
